# Training the domestic ferret to discriminate odors associated with wildlife disease

**DOI:** 10.1371/journal.pone.0259415

**Published:** 2021-11-01

**Authors:** Glen J. Golden, Maryanne Opiekun, Talia Martin-Taylor, Bruce A. Kimball

**Affiliations:** 1 Department of Biomedical Sciences, Colorado State University, Fort Collins, CO, United States of America; 2 Monell Chemical Senses Center, Philadelphia, PA, United States of America; PLOS, UNITED STATES

## Abstract

Recent avian influenza infection outbreaks have resulted in global biosecurity and economic concerns. Mallards are asymptomatic for the disease and can potentially spread AI along migratory bird flyways. In a previous study, trained mice correctly discriminated the health status of individual ducks on the basis of fecal odors when feces from post-infection periods were paired with feces from pre-infection periods. Chemical analyses indicated that avian influenza infection was associated with a marked increase of acetoin (3-hydroxy-2-butanone) in feces. In the current study, domesticated male ferrets (Mustela putorius furo) were trained to display a specific conditioned response (i.e. active scratch alert) in response to a marked increase of acetoin in a presentation of an acetoin:1-octen-3-ol solution. Ferrets rapidly generalized this learned response to the odor of irradiated feces from avian influenza infected mallards. These results suggest that a trained mammalian biosensor could be employed in an avian influenza surveillance program.

## Introduction

Avian influenza virus (AIV) has been identified for its potential to disrupt the economy of the poultry industry through devastating losses of farmed fowl [1,2]. Waterfowl and shorebirds are the natural reservoir of all subtypes of AIV, are distributed across the globe, and are considered primarily responsible for the spread and maintenance of AIV [1,2]. Infected wild waterfowl and shorebirds are typically indistinguishable from uninfected animals in the field.[1–3]. Furthermore, there is evidence that highly pathogenic (HP) AIV strains may arise after low pathogenic (LP) AIV are introduced to poultry from wild birds and subsequently mutate within poultry [4–6]. Given the potential impacts of AIV to domestic animals and human health, it is imperative that new, reasonably cost-effective tools be developed for AI detection.

There is now growing and extensive evidence that certain diseases can alter human and animal bodily odors. In recent studies, it was shown that bodily secretions, such as urine, contain volatile odorants that undergo quality or intensity changes that are detectable by trained mice following events such as immunization, inflammation, or brain trauma [7,8]. Furthermore, trained mice have been shown to correctly discriminate the health status of individual ducks on the basis of fecal odors when feces from post-viral infection periods were paired with feces from pre-viral infection periods [9]. Interestingly, chemical analyses indicated that avian influenza infection was associated with a marked increase of acetoin (3-hydroxy-2-butanone) in feces. This was shown as an increase in acetoin peak responses relative to 1-octen-3-ol responses (from 5:1 to over a 100:1) resulting from AIV infection and a ratio closer to 1:1 or 2:1 ratio from non-infected control animals.

Biosensors have been used in studies to discriminate various tissues manifesting cancer [10] from healthy tissue in a number of scientific studies; including lung, prostate, colorectal, ovarian, breast, bladder, and skin cancers and to detect, in samples of human sputum, the presence of *Mycobacterium tuberculosis* [11] which causes tuberculosis. Trained detector dogs have already been shown to be invaluable tools for wildlife research. Dogs have been employed for scat [12], carcass [13], and pest detection [14]. However, dogs with the behavioral attributes required of an animal that is not distractible in the field, can work long periods of time, and be able to work for a period of time that would justify the amount of training involved are quite rare and hard to find. Additionally, the expense of maintaining a dog colony can be prohibitive. Thus, we sought to use a “bridge” species that could replicate the individual response a dog might provide, but in a limited laboratory setting that does not offer the challenges of natural setting with its abundance of sensory stimuli. Additionally, everything the experimenters learned from training in the ferret study will be used to inform dog training in the next phase of this work.

We hypothesized that the success of the mice in detecting AIV in mallard fecal samples could be repeated in a species that had a more malleable behavioral repertoire (i.e., a proposed canine AI biosensor program). Although a study examining the aptitude of dogs in disease detection was desired, the cost and logistical factors proved to be prohibitive without the support of further evidence being provided. Ferrets were chosen as a “bridge” species between the mouse study and a potential canine AI biosensor program to further demonstrate detection of AIV-infected individuals. While ferrets were expected to work well in odor discrimination and detection tasks in the laboratory, it was anticipated that a natural setting would prove too great a challenge to a ferret’s ability to focus. Domestic ferrets were chosen for collecting further evidence in the laboratory because of their learning discrimination capabilities and dog-like social-cognitive skills in human interactions [15,16]. A previous study with ferrets demonstrated their ability to detect peppermint odor in multi-choice experiments [17]. In the current study, domesticated male ferrets (***Mustela putorius furo***) were trained to display a specific conditioned response (i.e. active scratch alert) in response to a marked increase of acetoin in a presentation of a acetoin:1-octen-3-ol solution. It may not be surprising; these same trained ferrets were able to discriminate infected from non-infected samples when confronted with irradiated feces from mallards experimentally infected with a low pathogenic avian influenza (LPAI) on the first attempt.

## Materials and methods

### Ethics statement

All biosafety precautions and experimental protocols were approved by the Monell Chemical Senses Center Institutional Animal Care and Use Committee (protocol number 1161).

### Biosensors

Eight, male, castrated ferrets (Marshall BioResources, North Rose, NY, USA) born 8/19-21/2013 arrived at Monell as juveniles at 15 weeks of age. Ferrets were housed in pairs in two level, 2.5 cm spaced 12 g wire cages (MidWest, Muncie, IN; 91.44 cm wide x 63.5 cm deep x 160.66 cm high) and maintained at 23°C on a 12-h light (12-h dark cycle). During food restriction feeding periods or in case of illness, the ramp that connected the upper and lower levels of the cage could be locked in a raised and closed position, allowing for each of the ferrets to be isolated. Environmental enrichment was provided both in the cages (blankets, hanging cubes, and hammocks) and during 60-minute free exercise periods daily on weekdays. Ferrets were given *ad libitum* access to tap water. Totally Ferret Complete diet (Performance Foods, Broomfield, CO, USA) was available *ad libitum* with the exception of food restriction periods during training and testing.

During food restriction periods, ferret weights were recorded every weekday prior to training, and their health was assessed every weekday (e.g., grooming, activity, visible signs of discomfort) before, during, and after training. Training or testing sessions lasted approximately 3.5 hours. Ferrets were also given *ad libitum* access to tap water during training or testing sessions. On days consisting of training or testing, food was provided after the session for 1 h while the ferrets were separated on different levels of the cage. Food bowls were weighed before and after the feeding session and the difference (i.e., mass of food assumed ingested) was recorded.

### Stimuli

Odorant compounds: *trans*-cinnamic aldehyde (cinnamaldehyde; CAS: 14371-10-9), vanillin (CAS: 121-33-5), acetoin (CAS: 513-86-0) and 1-octen-3-ol, (octenol; CAS: 3391-86-4) were technical grade (> 96% or better; Sigma-Aldrich, USA). Odorant concentrations were diluted from stock with propylene glycol (PG; CAS: 57-55-6) and prepared weekly. Ratios of acetoin:1-octen-3-ol were initially chosen based on a previous study of fecal odorants [9]. Duck feces were prepared by reconstituting 1 g of dessicated feces for each donor with 1 ml of distilled H_2_O in the glass training vial and the rehydrated fecal samples were stored a 4˚ C.

### Irradiated duck feces

Feces collected from eight farm-raised mallards of mixed gender were used from a prior study [9]. Briefly, in that study six ducks were infected with a low pathogenic strain of AIV (H5N2) in 2009. Eight pre-treatment and eight post-treatment samples were collected and stored frozen at -80˚ C. Samples from the six infected and two control mallards were inactivated by being subjected to 2.7 Mrad of cesium irradiation for 27 hours and lack of infectivity was confirmed prior to transport (see Kimball, Yamazaki et al. 2013 for details). The samples had been stored in a -80˚ C freezer for six years prior to use in this study.

### Ferret odor alert response and odor discrimination training

For an overall view of ferret training, please see Table 1. Ferrets were initially allowed a two-week acclimation period that included daily handling and daily 60-minute exercise periods (all eight ferrets were allowed loose on the testing room floor for interaction with each other, various toys, and the experimenters (GJG, MO, or TMT). To test the ferrets’ response to operant conditioning with the use of a hand-held “clicker”, ferrets were trained to stand on their hind limbs when presented with a hand signal (i.e., waving an outstretched hand from just above the heads of the ferret towards the ceiling) and a verbal cue (i.e., “Up”). A correct response was reinforced with the sound of the clicker and rewarded with a small amount of FerretVite (8 in 1, Spectrum Brands, USA), a high calorie vitamin supplement delivered from a 10-ml syringe fitted with modified stainless steel sipper tube. Incorrect responses were not rewarded and the experimenter turned away and walked to a new position in the room and another attempt was made. Once ferrets responded consistently (~100%) to this command (approximately 10 trials/ferret), shaping the ferrets’ odor alert response behavior was started.

**Table 1:** Overall view of training the ferrets from introducing clicker training to testing in-between ratios of acetoin: Octenol.

Ferret Biosensor Training Flow Chart		
Step	Description	Criteria for success/sessions to success
**1**	Training association of reward and the “clicker”	All ferrets standing on hind limbs with verbal cue
**2**	Introducing the “scratch” box	Ferrets willing to interact with the box
**3**	Shaping the odor alert response (i.e., scratching at the “scratch” box) to a single box	Ferrets willing to scratch with one or both forepaws at the box for reward
**4**	Shaping the odor detection response to one box (containing reward) out of five boxes (four without reward); 5 trials/session	80% accuracy at choosing box with FerretVite; 1 session
**5**	Shaping the odor detection response to one box (containing odor) out of five boxes (four without odor); 5 trials/session	80% accuracy at choosing box with cinnamaldehyde; 4 sessions
**6**	Shaping the odor discrimination response to one box (containing cinnamaldehyde) out of five (four containing a vanillin); 5 trials/session	90% accuracy at choosing box with cinnamaldehyde; 3 sessions
**7**	Training to alert to a high acetion:octenol ratio (1 box) in comparison to low acetion:octenol ratios (4 boxes) 5 trials/session for 8 sessions, then 10 trials/session	2 ferrets failed to meet 80% accuracy. Remaining 6 ferrets performing at 90% accuracy in choosing box with high acetion:octenol ratio; 2 sessions
**8**	Introduction of double blind procedure in discrimination between differing acetion:octenol ratios; 10 trials/session	80% accuracy at choosing box with high acetion:octenol ratio in at least one session; 14 sessions
**9**	Introduction of extinction trials (see text for definition of extinction); 10 trials/session	80% accuracy at choosing box with high acetion:octenol ratio; 4 sessions
**10**	Introduction of multiple box sets (n = 3) for use during training; 10 trials/session	80% accuracy at choosing box with high acetion:octenol ratio; 9 sessions
**11**	Increased number of trials/session to 12	80% accuracy at choosing box with high acetion:octenol ratio; 9 sessions
**12**	Increased number of box sets to 12; 12 trials/session	80% accuracy at choosing box with high acetion:octenol ratio; 9 sessions
**13**	Testing of novel ratios of acetion:octenol; 12 trials/session	—

### Scratch boxes

**Fig 1:**
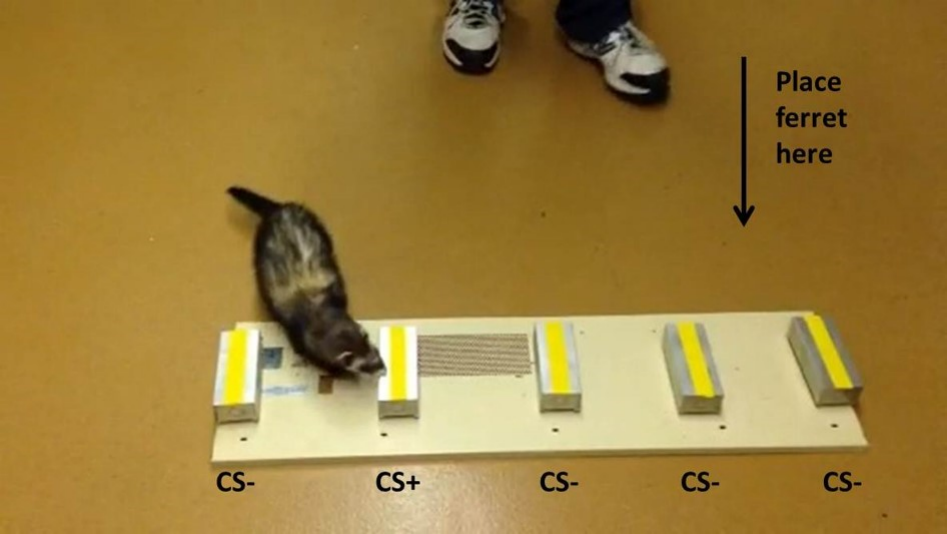
Apparatus to monitor operant-conditioned responses of trained ferrets to odors emitted from fecal samples derived from low pathogenic avian influenza (LPAI) infected and non-infected donor mallards. Aluminum “scratch” boxes were equipped with a sliding, self-locking cover and over 100 drilled holes (2-mm) in the cover and ends of the base compartment to allow for the escape of volatile odors. In the early stages of training, tape was used to modify the size of the drilled holes to prevent injury to the nails of the ferrets. The position numbers of the boxes were 1 through 5, left to right in all trials.

An aluminum “scratch” box (Ray Allen Manufacturing, Colorado Springs, CO, USA) was used to shape the odor alert response and for odor discrimination training and testing. The scratch box (14.6 cm x 5.7 cm x 3.8 cm) was equipped with a sliding, self-locking cover and over 100 drilled holes (2-mm) in the cover and ends of the base compartment to allow for the escape of volatile odors. Initially, tape was used to cover the drilled holes in order to prevent the small nails of the then still young ferrets from being caught and held in the hole. The tape was then punctured with an 18 G needle to conform to the drilled holes. This tape was removed and replaced during cleaning of the boxes. Use of the tape was discontinued following acetoin:octenol ratio training and testing as the nails of the ferrets had grown large enough that they could not catch in the drilled holes. A magnet was attached to the bottom of the base compartment allowing for attachment to a metal surface (recycled, metal panel; 94.6 cm L x 27.3 cm W with a 1.3 cm 45° lip bend on one of the long sides). Both the cover and base compartment were individually numbered with a permanent marker. The stimulus type or sample number was also written with a permanent marker on one end of the scratch box. In blind trials, this end was pointed away from the ferret handler so they were only visible to the box handler. The base compartment was fitted with a piece of egg crate lighting panel (cut to fit tightly) and customized to allow for the retention of a small 1 ml glass vial (Qorpak, Bridgeville, PA, USA). Vial caps (plastic septum-type screw caps with a 9 mm diameter opening) were fitted with 10 mm, Whatman qualitative filter paper, grade 1 (Sigma-Aldrich, USA) that allowed for the escape of volatiles, but prevented escape of samples placed in the vial. The scratch box was placed on the testing room floor during exercise and the ferrets were encouraged to investigate it thoroughly (Fig 1).

### Odor alert response shaping

The odor alert response was defined as the ferret scratching with both fore paws at the box. This response was shaped in steps. The first step (i.e., approach) was shaped by responding with a click and a reward (i.e., FerretVite) each time they approached the box. The box was allowed to stand freely on the linoleum floor so the ferrets would not be frightened by the sound of metal and the movement of the box in case these events were to occur once the boxes were fixed to a metal panel. Once each ferret behaved consistently (~100%) when the box was present, the reward sequence was withheld until the approaching ferret placed a forepaw on the box. Once the ferrets were each performing this behavior consistently (~100%), the reward was once again withheld until each ferret would scratch with both fore paws at the box. When a consistent response (~100%) to the presence of the box was achieved, a single scratch box was attached by its magnet to a recycled, metal panel (94.6 cm L x 27.3 cm W with a 1.3 cm 45° lip bend on one of the long sides).

Five scratch boxes were attached to the metal panel approximately 15.9 cm apart and in the center of the board (Fig 1). One randomly positioned box of the five on the panel had ~1 ml of FerretVite (CS+) on a plastic weigh boat (4.1 x 4.1 x 0.8 cm) attached with double sided tape to the egg crate inside. The remaining four boxes had the identical support system with no FerretVite in the weigh boat. Daily sessions consisted of 5 trials for each of the eight ferrets with the position of each box being pseudo-randomized for each trial. When an individual ferret was placed on the floor and alerted to the box with the reward inside, the sound of the clicker was presented and the box was opened by the experimenter to allow the ferret to lick the reward from the weigh boat. It is important to note that 20% was the level chance as only 1 of 5 boxes contained the reward. Food-restricted ferrets were able to perform this task at 80% accuracy or better (i.e., 83–100%) by day two of training. This training was performed for ~15 sessions to strengthen the odor alert response behavior. Following these sessions, the ferrets were given three sessions where the reward (i.e., FerretVite) was paired with a vial containing 1M cinnamaldehyde (CS+) in the same scratch box while the remaining 4 boxes remained empty. The position of each box was pseudo-randomized for each trial. Food-restricted ferrets were able to perform this task at 83% accuracy or better by day two of training.

Next, ferrets were asked to alert to the scratch box holding 1M cinnamaldehyde (CS+) alone while the remaining 4 boxes remained empty. Upon making a correct choice the ferrets received a reward delivered by the handler using a syringe. Again, daily sessions consisted of 5 trials for each of the eight ferrets with the position of each box being pseudo-randomized for each trial. Six individual ferrets were able to perform this task consistently at 80% or better after four sessions (or 20 trials) while two continued to struggle with the task as evidenced by their inability to choose the correct box. To see if contrast was needed for all ferrets to perform consistently, the remaining four boxes held 1 M vanillin for the next 5 sessions. By the third session, all ferrets were performing at accuracy rates of 80–100%. Finally, ferrets were asked to discriminate the box holding a lower concentration of cinnamaldehyde (0.1 M) from boxes holding 1 M vanillin over three sessions to determine if the ferrets would generalize their alert to a different concentration of the same learned odorant. Ferrets as a group performed at 90% or better from the first session for all three sessions.

### Shaping the behavior for discrimination of differing ratios of acetoin:1-octen-3-ol

In a previous study from our laboratory, chemical analyses demonstrated that LPAI infection was associated with a marked increase of 3-hydroxy-2-butanone (acetoin) in relation to 1-octen-3-ol (octenol) in feces. The ratio of these compounds was significantly greater in feces of infected ducks (10–50:1) in comparison to ducks pre-infection (1–5:1). Having replicated that ferrets could perform single compound detection [17], the ability to discriminate between single compounds was explored. Furthermore, we sought to determine if ferrets could discriminate among odor ratios and if they would generalize to novel ratios (differing from those with which they were trained). Eight ferrets given the opportunity to alert to a single scratch box holding a single vial of acetoin:octenol (randomly chosen from among 10:1, 20:1, 30:1, 40:1, or 50:1) presented in association with four scratch boxes holding vials of vehicle (i.e., PG). There were five trials (one of each ratio presented in pseudorandom order) for each session. After 8 sessions, the ferrets were performing at 80% accuracy as a group. The numbers of trials were then increased to determine if the ferrets could perform more trials within a single session. For two sessions, six ferrets were asked to alert during 10, rather than 5 trials. Two ferrets were also removed from the experiment, because they failed to meet the minimum criterion of 80%. The remaining six ferrets were performing at 90% accuracy by the second session. These six ferrets were used for the remainder of this study.

Next, the ferrets were asked to alert to a single, high acetoin:octenol ratio (10:1, 20:1, 30:1, 40:1, or 50:1) reflective of feces from LPAI infected ducks (CS+) in the presence of low acetoin:octenol ratios (1:1 or 2:1) reflective of feces from non-infected ducks (CS-). Each panel presented to the ferrets consisted of one scratch box with a randomly selected high acetoin:octenol ratio solution vial (CS+), two 1:1 acetoin:octenol ratio solution vials, and two 2:1 acetoin:octenol ratio solution vials (all CS-). The position of each box was pseudo-randomized for each trial. After 5 sessions (50 trials for each ferret), each ferret had performed at 60% or better (as high as 90%) accuracy and were consistently performing at 60% or better as a group. Again, it is important to note that 20% was the level chance as only 1 of 5 boxes contained the CS+ ratio.

### Double blind procedure

To avoid the possibility of the handler inadvertently communicating the position of the CS+ to the ferret, a double-blind procedure was incorporated into the training regimen employing a separate “box handler” and “ferret handler”. The box handler designed the daily schedule, consisting of the position of the CS+ scratch boxes, the order (i.e., trial number) of CS+ ratio presentation, and the order the ferrets were to perform their individual sessions. The box handler positioned the CS+ and CS- scratch boxes on the board, placed the board on the ground to signal the start of a trial, confirmed or rejected the ferret handler’s call (described in the next sentence) and positioned the boxes on the board for the next trial. The ferret handler controlled when the ferrets were to start a trial and called out “Hit” when the ferret made a decision and alerted to one of the boxes. The box handler responded “correct” or “incorrect” (based on the box handler’s knowledge of CS+ location) so that the ferret handler could rapidly react appropriately to the ferret’s decision. For a correct response, the ferret handler clicked the clicker and provided a small amount of FerretVite with a modified syringe. When the box handler rejected the ferret handler’s call of the alert, the ferret handler picked up the ferret and faced away from the box handler as the box handler picked up the board and prepared it for the next trial. This method was used for all remaining sessions, including additional shaping of behavior, training, and experimental testing.

### Blind discrimination of differing ratios of acetoin:1-octen-3-ol

Blind discrimination was performed as described above. After 14 sessions (140 trials for each ferret), each ferret had performed at 80% or better in at least one session (as high as 100%) accuracy and were consistently performing at 60% or better (as high as 73%) as a group, but this seemed to be the maximum accuracy they would attain. To improve individual accuracy, extinction trials were introduced into each session. We define an extinction trial as a trial consisting of CS+ and CS- samples that are familiar to the ferret (i.e., in terms of the quality, intensity, or donor identity) but is not rewarded or acknowledged in any way. The board was simply picked up immediately after a box selection, regardless if it was a correct or incorrect choice. We incorporated 3 random extinction trials during each session with the following conditions: extinction trials could not occur during the first or last trials and could not occur in two consecutive trials. Each ferret performed at 80% or better (as high as 100%) accuracy and as a group were consistently performing at 80% by the fourth session and 87% by the fifth session. We then added an additional extinction trial for a total of four per session out of 10 trials for three sessions with similar accuracy results.

### Number of box sets

Up to this point, we employed one set of CS- scratch boxes and a different scratch box for each of the CS+ stimuli. To control for the possibility that the ferrets were alerting to the box itself, we began to use three sets of scratch boxes for the CS- stimuli and continued using different scratch boxes for each CS+ stimulus. At this point we also increased the number of trials from 10 per session to 12 per session in order to better accommodate the four extinction trials in each session. There was an immediate drop in accuracy to 53% as a group. While this was still above chance, it did suggest that the identity of the box or the increased number of trials was, in some way, contributing to the choice being made by the ferrets. Scratch boxes were not dedicated to a particular valence and the valence of a particular box was changed frequently (i.e., boxes used the positive conditioned stimulus [CS+] were later used for the negative conditioned stimulus [CS-] and vice versa after washing). Thus, it was unlikely for CS+ boxes to “acquire a definitive signal” over time because these same boxes had the same chance of being a CS- over the sessions. By the 9th session, the ferrets were performing at 81% accuracy as a group and between 58–92% accuracy as individuals. To ensure that scratch box identity was no longer being used (or at least, its importance greatly diminished) as a component of the odor discrimination, we began using a new set of boxes for every trial in a session. The results are shown in Table 2.

**Table 2:** Training sessions using 12 box sets (1 box set of 5 boxes per trial) with all rewarded trials or 12 box sets and extinction trials included.

	Individuals			Overall		
Experiment	Rewarded	Extinction	Total	Rewarded	Extinction	Total
**Number of trials**	48	--	--	288	--	288
**12 box sets, all rewarded trials**	67–100%	--	--	83%	--	83%
**Number of trials**	60	20	80	360	120	480
**12 box sets, rewarded and extinction trials**	82–95%	80–95%	--	89%	88%	89%

### Blind discrimination of novel ratios of acetoin:1-octen-3-ol

To determine if the ferrets would be able to generalize to ratios they had not experienced previously, we introduced non-rewarded generalization trials. We define non-rewarded generalization trials here as trials conducted with CS+ stimuli unique in quality, intensity, or donor identity but having the same consequences as an extinction trial (i.e., the panel and boxes are picked up from the floor with no behavioral consequence for the ferret). Because the ferrets experienced a neutral response from the handler immediately following a generalization trial, it was assumed that little or no learning occurred during a non-rewarded generalization trial. Two novel ratios (25:1 and 35:1 acetoin:octenol) were randomly presented during the four non-rewarded generalization trials. Results are shown in Table 3.

**Table 3:** Testing sessions including generalization trials of in-between ratios of acetoin: Octenol reflecting AIV infected individuals.

Overall				
Experiment	Rewarded	Generalization	Generalization	Total
**Test ratio**		**25:1**	**35:1**	
**Number of trials**	144	36	36	216
**12 box sets**	92%	92%	83%	91%

Next, the ferrets’ responses to 3:1 and 5:1 acetoin:octenol ratio solutions (twice each) were randomly presented in four non-rewarded generalization trials. These acetoin concentrations represent borderline ratios very near the critical ratio delineating LPAI infected and non-infected conditions (see Table 5 in Kimball, Yamazaki et al. 2013). Results are shown in Table 4.

**Table 4:** Testing sessions including generalization trials of in-between ratios of acetoin:octenol reflecting non-infected individuals.

Experiment	Rewarded	Generalizations	Generalizations	Total
**Test ratio**		**3:1**	**5:1**	
**Number of trials**	144	36	36	216
**12 box sets**	83%	75%	67%	81%

### Experiment 1—Generalization to irradiated duck feces

To determine if trained ferrets (n = 6) would generalize the training they received with acetoin:octenol ratio solutions to actual fecal samples collected from LPAI infected and non-infected mallards, we presented the ferrets with irradiated duck fecal samples from a previous study. The CS- samples consisted of randomly chosen fecal samples collected pre-infection from all eight mallard donors for the Kimball et al., study (2013). Because of the low number of available samples, post-infection feces had to be presented in both rewarded and unrewarded (generalization) trials. The first two days of sessions included only rewarded trials followed by three days of double blind test sessions with unrewarded generalization trials. Daily testing session included four generalization trials randomly presented among twelve trials per session resulting in 72 overall unrewarded generalization trials.

### Data analysis

Training session results (correct or incorrect selection of the CS+ sample) were first subjected to Cochran-Mantel-Haenszel (CHM) test of general association to determine if correct rates of CS+ identification differed between sessions or among ferrets. Similarly, results of the rewarded trials during the three testing sessions were subject to CHM test. Cumulative responses of all ferrets across all appropriate sessions were calculated for: all training trials, rewarded trials during testing sessions, and generalization trials. Success rates (number of correct trials divided by the total number of generalization trials) were subjected to statistical tests of binomial proportion and Wilson confidence intervals were determined (proc FREQ in SAS). Null hypotheses differed for each cumulative score. For training trials, the hypothesis that cumulative score was greater than 80% was tested. For testing trials, the hypotheses that cumulative scores were greater than 75% for rewarded trials and 20% for generalization trials were tested. Cumulative scores for testing were anticipated to show a slight reduction in comparison to training scores as the stimulus being tested was novel.

Incorrect selections were further examined to determine if design components influenced box selection. Chi-square tests of association were conducted to determine if any particular CS+ donor (mallard), CS+ box position (1 thru 5 from left to right in the box array), or CS- donor (mallard) may have resulted in an incorrect selection.

## Results

### Experiment 1—Generalization to irradiated duck feces

**Fig 2:**
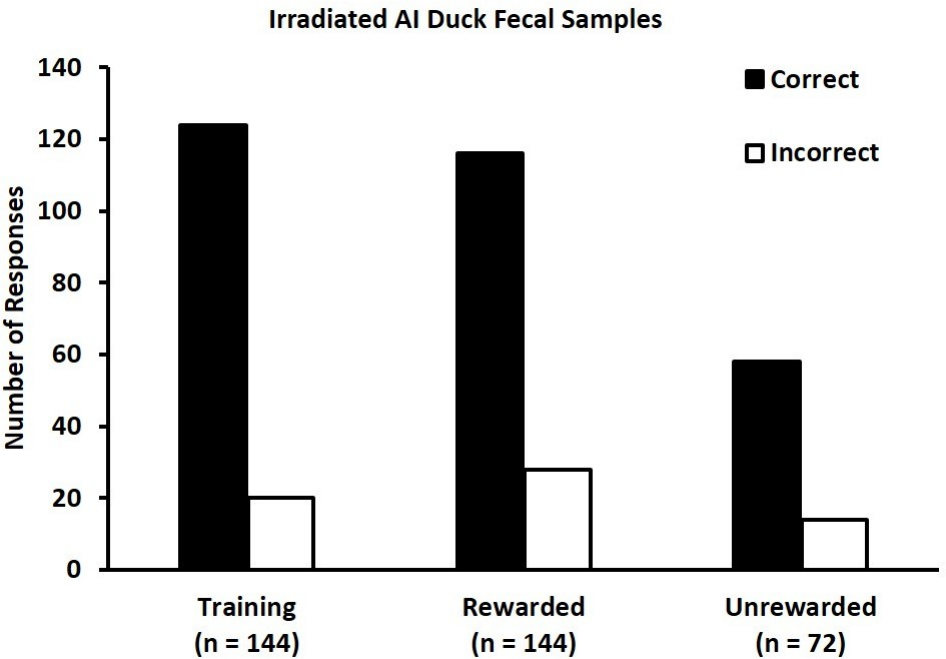
Ferrets can identify irradiated LPAI infected fecal samples in comparison to irradiated pre-infection and non-infected fecal samples. During training, ferrets correctly (black bar; left bars) identified the location of the single fecal sample derived from an LPAI infected donor with 86% accuracy, which was greater than 80% (p = 0.033). During rewarded testing trials, ferrets correctly (black bar; middle bars) identified the location of the CS+ samples with 81% accuracy, which was statistically similar to 75% (p = 0.062). During unrewarded generalization trials, ferrets correctly identified (black bar; right bars) the CS+ samples with 80% accuracy, which was significantly greater than the null hypothesis of 20% (p < 0.0001). White bars represent incorrect choices.

In two days of rewarded training trials, identification of the CS+ sample did not differ between sessions or among the six ferrets (p = 0.993). Collectively, ferrets correctly identified the location of the single fecal sample derived from an LPAI infected donor with 86% accuracy, which was greater than 80% (Fig 2; p = 0.033).

During testing sessions, responses in rewarded trials did not differ among sessions or ferrets (p = 0.702) and the ferrets collectively identified the location of the CS+ samples with 81% accuracy, which was statistically similar to 75% (Fig 2; p = 0.062). Unrewarded generalization trials also were consistent across sessions and ferrets (p = 0.732) and collectively identified the CS+ samples with 80% accuracy, which was significantly greater than the null hypothesis of 20% (Fig 2; p < 0.0001). Examination of incorrect trials indicated that neither the identity of CS+ donor (p = 0.108), CS- donor (p = 0.227), or box position of CS+ sample (0.787) influenced ferret identification of CS+ samples.

## Discussion

This study provides evidence that ferrets are capable of identifying irradiated feces from LPAI-infected mallards immediately after training with solutions of two key odorants (Fig 2). Interestingly, on the very first trial the ferrets were exposed to the irradiated fecal samples, they each explored the boxes thoroughly and walked past the CS+ box at first regardless of its position on the panel. However, before reaching the next box or turning to go back along the boxes, each ferret quickly returned to the prior box to alert at the scratch box containing the CS+ sample correctly.

The level of food restriction experienced by the ferrets during training and testing, either by time or volume, has been shown to be required for reliable operant conditioning responses in rats and minimally stressful in terms of behavior, appearance and physiology [18–20] in that species. Ferrets have also been shown to respond to operant conditioning tasks reliably with this level of restriction with little or no signs of stress [17]. Though the ferrets were not water restricted, food restriction is known to result in a decrease in water intake. The ability of rodents to acclimate to water restriction has been attributed to efficient reduction of fluid and energy loss through behavioral and physiological adjustments [19–21].

The result of this experiment provides clear-cut evidence that ferrets are not only capable of performing a complex olfactory discrimination task but can be utilized to perform non-invasive detection of waterfowl LPAI status. These results confirm the results of a previous experiment on the ability of a biosensor to detect the presence of LPAI infection at a high rate of accuracy: Mice trained as biosensors demonstrated 76% accuracy in choosing a y-maze arm associated with fecal odors from infected ducks during generalization trials [9] and ferrets demonstrated 81% accuracy for detecting fecal odors from LPAI infected ducks during generalization trials from a panel that also included four fecal odors from non-infected ducks (Fig 2) in the present study.

One hypothesis to explain these results is that the ferrets generalized the odor cue learned during acetoin:octenol ratio training to fecal odors from LPAI infected samples [22,23]. Another explanation could be that the ferrets adopted a discrimination strategy to identify successfully the single odor source that differed from the others. This strategy requires no past experience or specific knowledge of the odors, only the ability to discriminate. The training with cinnamaldehyde in one box and four empty boxes versus training with cinnamaldehyde versus vanilla shows evidence that suggests that it seemed easier for the ferrets to perform discrimination rather than simple detection. Single trial learning is well established in the conditioned taste and odor aversion literature in both humans [24] and rodents [25]. Furthermore, there is strong evidence for single trial learning appetative odor stimulus models [26,27]. Therefore, it is not unreasonable to believe that ferrets learned new odor cues quickly despite not being rewarded with the secondary reinforcer (i.e., the sound of the clicker) or the actual reward itself in experiments where novel stimuli were presented in unrewarded generalization trials.

However, this rapid learning hypothesis would not be sufficient to explain why all six ferrets were able to identify the LPAI infected sample during the first trial of the first session,. even when the CS+ was in the first box of the panel and no other boxes were sampled. This observation could suggest that the odor cue the ferrets use to identify the infected sample is a change in acetoin intensity or a change in the ratio of acetoin in relation to octenol or some other compound and not a case of choosing the only sample that was unlike the remaining samples. In addition, there are other, less likely, odor cues the ferrets could have learned about without the investigators even being aware of, although we did our best to control for these variables (i.e., box identity, previous valence of box, etc.).

Fecal matter from waterfowl contain viable avian influenza virus. Thus, waterfowl and waterfowl habitats are the central source of avian influenza virus in all other species, including farm raised fowl [1,28]. Current surveillance methods include collecting cloacal swabs from collected waterfowl. Cloacal swabs could then be presented to a trained biodetector as an initial screening step. This makes fecal sampling in the wild an integral part of any surveillance system designed for the early detection of avian influenza viruses. Fecal sampling in the wild is economically reasonable, although the samples collected must be fresh and usually contain some form of contaminant [1]. Our current results suggest that the use of biosensors trained to detect and identify fecal matter derived from waterfowl infected with avian influenza viruses would add a layer of surveillance to the current system that exhibits even greater efficiency by offering a method to limiting areas of wetlands that would need to be sampled.

The convenience of training ferrets with acetoin:octenol solutions made conversion to training with duck feces very easy and suggests that a surveillance program consisting of trained biosensors can be developed without need for training with hazardous biological samples. However, in forcing the ferrets to focus on changes in acetoin intensity, did the training and experimental design force them to focus on the acetoin and ignore a potentially superior cue that was not revealed by chemical analysis? This study clearly demonstrates the feasibility of deploying trained biosensors for avian influenza virus surveillance in waterfowl.

This experiment provides further evidence that a signature odor results from LPAI. However, there are still important questions to be answered. For example, was fecal irradiation necessary to produce the signature odor of AI infection? Since ferrets are susceptible to influenza A virus infections, what is the likelihood that a ferret become infected with influenza A virus after being used as biosensors to detect acetoin and exposed to IAV in feces when sniffing/inhaling the fecal odors? Can a biosensor differentiate between samples that result from infection with different types of respiratory viruses producing gastrointestinal effects? Most importantly, can a biosensor trained to identify LPAI in mallard fecal samples identify the odor identity of avian influenza infection in another species?

The likelihood remains very high that detection and affirmation of avian influenza infection will be as successful in canine biosensors. Domestic ferrets show skill in learning discrimination tasks and dog-like social-cognitive skills in interacting with humans [15,16] and there are numerous examples in the literature describing the high accuracy of dogs in detecting disease via the sampling of various modes and tissue types (i.e., urine, skin, breath, etc.) [29,30].
